# Subtle mutations in the *SMN1* gene in Chinese patients with SMA: p.Arg288Met mutation causing *SMN1* transcript exclusion of exon7

**DOI:** 10.1186/1471-2350-13-86

**Published:** 2012-09-20

**Authors:** Qu Yu-jin, Du Juan, Li Er-zhen, Bai Jin-li, Jin Yu-wei, Wang Hong, Song Fang

**Affiliations:** 1 Department of Medical Genetics, Capital Institute of Pediatrics, Beijing, China; 2 Department of Neurology, Children’s Hospital Affiliated Capital Institute of Pediatrics, Beijing, China

**Keywords:** Spinal muscular atrophy, Survival motor neuron gene-1, Subtle mutation, Transcript

## Abstract

**Background:**

Proximal spinal muscular atrophy (SMA) is a common neuromuscular disorder resulting in death during childhood. Around 81 ~ 95% of SMA cases are a result of homozygous deletions of survival motor neuron gene 1 (*SMN1*) gene or gene conversions from *SMN1* to *SMN2*. Less than 5% of cases showed rare subtle mutations in *SMN1*. Our aim was to identify subtle mutations in Chinese SMA patients carrying a single *SMN1* copy.

**Methods:**

We examined 14 patients from 13 unrelated families. Multiplex ligation-dependent probe amplification analysis was carried out to determine the copy numbers of *SMN1* and *SMN2*. Reverse transcription polymerase chain reaction (RT-PCR) and clone sequencing were used to detect subtle mutations in *SMN1*. *SMN* transcript levels were determined using quantitative RT-PCR.

**Results:**

Six subtle mutations (p.Ser8LysfsX23, p.Glu134Lys, p.Leu228X, p.Ser230Leu, p.Tyr277Cys, and p.Arg288Met) were identified in 12 patients. The p.Tyr277Cys mutation has not been reported previously. The p.Ser8LysfsX23, p.Leu228X, and p.Tyr277Cys mutations have only been reported in Chinese SMA patients and the first two mutations seem to be the common ones. Levels of full length *SMN1* (fl-*SMN1)* transcripts were very low in patients carrying p.Ser8LysfsX23, p.Leu228X or p.Arg288Met compared with healthy carriers. In patients carrying p.Glu134Lys or p.Ser230Leu, levels of fl-*SMN1* transcripts were reduced but not significant. The *SMN1* transcript almost skipped exon 7 entirely in patients with the p.Arg288Met mutation.

**Conclusions:**

Our study reveals a distinct spectrum of subtle mutations in *SMN1* of Chinese SMA patients from that of other ethnicities. The p.Arg288Met missense mutation possibly influences the correct splicing of exon 7 in *SMN1*. Mutation analysis of the *SMN1* gene in Chinese patients may contribute to the identification of potential ethnic differences and enrich the *SMN1* subtle mutation database.

## Background

Proximal spinal muscular atrophy (SMA) is a common infantile neuromuscular disease with an incidence of 1 in 6000 ~ 10,000 among newborns [1]. It is characterized by the destruction of alpha motor neurons in anterior horn cells of the spinal cord. This leads to progressive symmetrical limb and trunk muscle weakness, along with atrophy [2]. This disease can be categorized into four clinical types (SMA I–IV) based on age at onset and maximum attained motor functions [3].

The SMA-determining gene, survival motor neuron (*SMN*), is located at chromosome 5q11.2-13.3 [4], with two almost identical copies: telomeric *SMN1* (MIM#600354; GenBank: NM_000344) and centromeric *SMN2* (MIM#601627; GenBank: NM_022875). *SMN1* and *SMN2* sequences are highly homologous with only five nucleotide differences, one in intron 6, one in exon 7, two in intron 7, and one in exon 8 [4]. Although these differences do not alter the encoded amino acids, *SMN1* produces mainly full-length *SMN* transcripts. However, 90% of the transcripts produced by *SMN2* exclude exon 7 owing to a conversion (C→T) at position 840 in exon 7 [5]. Mutations in *SMN1* result in the SMA phenotype, whereas *SMN2* copy numbers determine the severity of SMA.

The majority of SMA patients have been found to have a homozygous deletion in exon 7 of *SMN1*. Some SMA cases are caused by compound mutations, with a *SMN1* deletion on one allele and a subtle mutation on the other. Since the first several mutations were identified at 1995 by Lefebvre *et al.*[4] and Bussaglia et al. [6], More than 60 subtle mutations of *SMN1* gene have been continuously identified worldwide [7-24]. Although mutations are distributed along the entire coding sequence of *SMN1*, the majority are located in exons 3 and 6 (47.5%). Tsai et al. reported the first subtle mutation in *SMN1* of Chinese SMA patients in 2001 [18]. To date, eight subtle mutations have been successfully identified in Chinese SMA patients [18-23]. In this article, our aim was to identify subtle mutations in *SMN1* of Chinese SMA patients and detect the *SMN1* transcript levels of these patients based on the quantitative RT-PCR.

## Methods

### Patients and materials

Fourteen SMA patients and their parents, from 13 unrelated families, were enrolled in this study. All patients met the diagnostic criteria of proximal SMA, with their clinical data provided in Table 1. Of these cases, three were diagnosed as type I SMA, nine were type II SMA, and two were type III SMA. Case 9 was the younger sister of case 8. Genomic DNA and total RNA from these individuals were isolated from peripheral venous blood using phenol-chloroform extraction and an RNeasy Kit (Qiagen, Germany), respectively. Samples of the parents of cases 1 and 7 were not available. This study was approved by the Ethics Committee of the Capital Institute of Pediatrics, and informed consent was obtained from all subjects.

**Table 1:** **Genotype and phenotype in patients with a subtle mutation of *SMN1 *gene**

**Family No.**	**Case No.**	**PhenoType**	**Gender**	**Age at last examination**	**Age of onset**	**Attained motor function**			***SMN1 *genotype**		**Point Mutationlocation**	***SMN2 *copies**	***Fl-SMN2 *transcript**	***Fl-SMN *transcript**	**Parental origin**
						**Head control**	**Sit unsupported**	**Walk independently**	**Allele1**	**Allele2**					
1	1	I	M	3y6m	4 m	+	-	-	Deletion	p.Ser8LysfsX23	Exon 1	2	-	-	ND
2	2	II	M	1y7m	11 m	+	+	-	Deletion	p.Ser8LysfsX23	Exon 1	2	6.33 ± 1.72	6.76 ± 1.94	Paternal
3	3	II	M	6y8m	1y2m	+	+	-	Conversion	p.Ser8LysfsX23	Exon 1	3	20.36 ± 11.09	21.01 ± 10.4	Paternal
4	4	II	M	5y	11 m	+	+	-	Deletion	p.Glu134Lys	Exon 3	2	7.73 ± 5.44	12.46 ± 5.47	Paternal
5	5	II	F	2y6m	1y1m	+	+	-	Deletion	p.Glu134Lys	Exon 3	2	6.41 ± 5.78	11.21 ± 3.45	Paternal
6	6	I	F	2y3m	4 m	+	-	-	Deletion	p.Leu228X	Exon 5	2	9.24 ± 6.6	10.277 ± 6.8	Maternal
7	7	II	M	4y1m	8 m	+	+	-	Conversion	p.Leu228X	Exon 5	3	-	-	ND
8	8	II	M	14y	10 m	+	+	-	Deletion	p.Ser230Leu	Exon 5	2	8.04 ± 7.27	12.22 ± 7.07	Paternal
	9	III	F	9y8m	2y	+	+	+†	Deletion	p.Ser230Leu	Exon 5	2	9.35 ± 5.74	14.83 ± 4.45	Paternal
9	10	II	M	6y4m	1y	+	+	-	Deletion	**p.Tyr277Cys§**	Exon 6	2	4.53 ± 3.47	6.54 ± 4.33	Maternal
10	11#	I	F	2y10m	5 m	+	-	-	Conversion	p.Arg288Met	Exon 7	3	13.1 ± 10.4	13.71 ± 10.6	Paternal
11	12#	II	F	4y4m	1y6m	+	+	-	Conversion	p.Arg288Met	Exon 7	3	9.46 ± 7.84	12.46 ± 8.01	Paternal
12	13	II	M	5y	10 m	+	+	-	Deletion	-	-	2	8.02 ± 1.97	9.86 ± 2.01	ND
13	14	III	M	15y	1y5m	+	+	+†	Conversion	-	-	3	21.67 ± 11.0	25.17 ± 11.8	ND

Conversion means deletion of *SMN1 *owing to conversion of *SMN1 *sequences to *SMN2* with the copy number of *SMN2 *increasing. *ND*, not detected. **§**Novel mutation; #,these patients had been reported [22]; †, Walk in waddling gait ;case 8 and case 9 are siblings.

### Analysis of subtle mutations in *SMN1*

Cloning and sequencing of reverse transcription polymerase chain reaction (RT-PCR) amplicons was carried out to analyze subtle mutations. First-strand cDNA synthesis was performed with 0.5 μg of total RNA, random primers, and M-MLV Reverse Transcriptase (Invitrogen, USA) in accordance with the manufacturer’s instructions. Specific PCR primers (SMN575 [13] and 541C1120 [4]), were used to amplify the *SMN* gene (exons 1–8) using LA Taq polymerase (TAKARA, Japan) and cDNA template. Thermal cycling conditions involved an initial denaturation step for 5 min at 94°C, followed by 30 cycles of 45 s at 94°C, 50 s at 60°C, and 60 s at 72°C, with a final extension step at 72°C for 10 min. Amplicons were subcloned into the pGEM-T Easy cloning vector (Promega, USA) according to the supplier’s protocol. *SMN1*and *SMN2* subclones were differentiated using restriction enzymes (*Dra*I and *Dde*I) [25]. Around 5 ~ 8 *SMN1* and 2 ~ 3 *SMN2* clones for each case were sequenced. Mutations were further confirmed by direct sequencing of the amplified products from *SMN* genomic DNA samples.

### Multiplex ligation-dependent probe amplification (MLPA) analysis

MLPA analysis was performed to detect copy numbers of *SMN1* and *SMN2* in all cases using a SALSA MLPA kit (P021-A1; MRC-Holland, Amsterdam, The Netherlands) according to the manufacturer’s recommendations. This SALSA kit contained 16 probes specific for the SMA critical region (5q12.2–q13.3). Among these, two specific probes for the C→T transition in exon 7 (C for *SMN1* and T for *SMN2*) and two specific probes were for the G→A transition in exon 8 (G for *SMN1* and A for *SMN2*) were included. In addition, the SALSA kit contained 21 control probes mapping to other autosomes. After MLPA treatment, products were run on the ABI 3730 automatic sequencing system (Applied Bio-Systems, USA). Four healthy individuals carrying two *SMN1* copies were the normal controls, with eight carriers (parents of the patients with a homozygous *SMN1* deletion) as the single copy *SMN1* reference. For each sample, raw data [relative peak area (RPA)] were analyzed and compared with normal controls using Gene marker version 1.75 software. This software is able to calculate the RPA for each probe and to compare RPAs with those derived from normal controls. All samples were analyzed at least twice. A ratio with normal controls in the range 0.7–1.3 indicated a normal copy number (two copies), a ratio less than 0.7 indicated one copy, a ratio between 1.3–1.6 was indicative of three copies, and a ratio equal to 0 indicated zero copy.

### Analysis of the novel p.Tyr277Cys mutation

The allele-specific primer, Y277CR (5′-CTG AGT GAT TAC TTA CCA TAC-3^′^), was designed to identify only the mutant allelic sequence, and was coupled with the upstream primer SMNE6F [12]. This was applied in an allele-specific PCR (AS-PCR) to screen for the p.Tyr277Cys mutation in 150 control individuals. Simultaneously, alignment analysis of SMN proteins from six different species was performed using Clustal X version 1.8 to analyze levels of conservation for tyrosine 277.

### Restriction endonuclease digestion of *SMN* transcripts

Total RNA (0.5 μg) was isolated from the peripheral blood of patients and controls, and used to synthesize first-strand cDNA as described earlier. The cDNAs were amplified using primers SMN575 [13] and 541C1120 [4] in a 50-μl reaction with a 60°C annealing temperature for 30 cycles as the section of “Analysis of subtle mutations in *SMN1* ”described. In general, *SMN* transcripts yielded three products, full-length *SMN1* (fl-*SMN1*, 1259 bp), full-length *SMN2* (fl-*SMN2*, 1259 bp) and *SMN2* isoform lacking exon 7 (Δ7-*SMN2*, 1205 bp). The *SMN1* transcripts could be distinguished from *SMN2* transcripts by digestion with the restriction enzyme *Dde*I. Following digestion, there was a 1259-bp fragment corresponding to fl-*SMN1*, a 1136-bp fragment corresponding to fl-*SMN2*, a 1082-bp fragment indicative of Δ7-*SMN2* and a 123-bp fragment from *SMN2*. The transcripts and their products after *Dde*I digestion were separated on a 6% polyacrylamide gel at 500 volts for 2 h. Gels were stained with silver stain.

### Quantitative RT-PCR (qRT-PCR)

Three plasmids (fl-*SMN1*, fl-*SMN2* and *GAPDH*) were constructed as external standards. Amplified *SMN1* and *SMN2* were obtained using normal control cDNA and primers SMN-F (5^′^-GCT GAT GCT TTG GGA AGT ATG TTA-3^′^) and SMN-R (5′-TCA ACT GCC TCA CCA CCG TGC TGG-3′), specific for exons 6 and 8, respectively. The primer pair for amplification of *GAPDH* was GAPDH_exst-F and GAPDH_exst-R, as previously described [26]. Amplicons for *SMN1*/*SMN2* (395 bp) and *GAPDH* (133 bp) were cloned into the pGEM-T Easy cloning vector (Promega, USA). Cloned inserts were all verified by sequencing. Plasmid DNA was extracted and quantified by absorbance using a NanoDrop 2000 (Thermo, USA). Based on plasmid length and concentration, the copy number of each plasmid can be calculated. These plasmids were serially diluted across a range (10^3^–10^8^ copies) and used as external standards to construct the standard curve.

A qPCR assay to quantify *SMN* transcripts was conducted as described by Tiziano et al. [26]. The primers and MGB-probes were designed using Primer Express v1.5 software (Applied Biosystems, USA), with sequences provided in Table 2. The fl*-SMN1* and fl-*SMN2* transcripts were amplified using the same primer pair (SMN_mgb-F and SMN_mgb-R). Full-length transcripts of the two genes were distinguished by two different Taqman MGB probes on the basis of the C→T transition located in exon 7. For *GAPDH*, the primers (GAPDH_abs-F and GAPDH_abs-R) and MGB probe sequence were the same as those described in Tiziano *et al.*[26] described. All reactions (20 μl) were carried out using a 7500 Real-Time PCR System (Applied Biosystems, USA) and contained 2× GoldStar TaqMan Mixture (KANGWEI, China), 20 ng of cDNA, 0.4 μl of each primer (10 pmol/μl), and 4 pmol of the *SMN1*, *SMN2* or *GAPDH* probe. The thermal cycling conditions involved 2 min at 50°C then 10 min at 95°C, followed by 40 cycles of 15 s at 95°C and 1 min at 60°C. Each sample was assayed in duplicate and repeated at least twice. Evaluation of data was performed using 7500 Software SDS version 1.4.

**Table 2:** Information of Primers and Probes Used for qRT-PCR

**Fragment**	**Primers**	**Probe**	**Amplify length**
SMN1	SMN_mgb-F: 5^′^TGGTACATGAGTGGCTATCATACTG3^′^	5^′^FAM-ATGGGTTT**C**AGAA-MGB-NFQ	75 bp
	SMN_mgb-R: 5^′^ GTGAGCACCTTCCTTCTTTTT3^′^		
SMN2	SMN_mgb-F: 5^′^TGGTACATGAGTGGCTATCATACTG3^′^	5′FAM-ATGGGTTT**T**AGAA-MGB-NFQ	75 bp
	SMN_mgb-R: 5^′^ GTGAGCACCTTCCTTCTTTTT3^′^		
GAPDH[26]	GAPDH_abs-F:5^′^GGGTGTGAACCATGAGAAGTATGA-3^′^	5′FAM-CAAGATCATCAGCAATGC-NFQ3^′^	73 bp
	GAPDH_abs-R: 5^′^CTAAGCAGTTGGTGGTGCAGG-3^′^		

The bases **C **and **T **in bold are specific for *SMN1 *and *SMN2*, respectively. The primers and probe sequences of GAPDH are described by Tiziano FD et al. [26].

### Statistical analysis

Transcript levels for fl*-SMN1*, fl-*SMN2* and *GAPDH* were expressed as copies per nanogram of total RNA. Expression levels of fl*-SMN1*, fl*-SMN2*, and fl*-SMN* were normalized to *GAPDH*. Statistical analysis was carried out using SPSS 19.0. A parametric test (*t*-test) was used to compare the transcript levels between normal controls and carriers, as well as between carriers and patients. Correlation between *SMN2* gene copy numbers and fl*-SMN2* transcript levels were analyzed by a gerneral linear model(one-way ANOVA). A *p*-value less than 0.05 was regarded as significant.

## Results

### *SMN1* and *SMN2* copy numbers

The numbers of *SMN1* and *SMN2* copies in the 14 patients analyzed by MLPA are presented in Table 1. All patients carried only one copy of *SMN1*. Patients 3, 7, 11, 12, and 14 carried three copies of *SMN2*. The remaining patients had two copies of *SMN2*.

### *SMN1* subtle mutation

**Figure 1:**
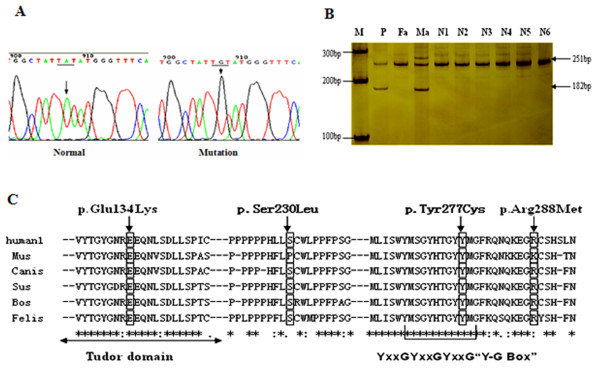
**Analysis of the novel p.Tyr277Cys mutation. **(**A**) Sequencing map of the p.Tyr277Cys (TAT > TGT) mutation. Underlined bases indicate codons affected by subtle mutations. Black arrows indicate the converted base peak. (**B**) AS-PCR screening results for the p.Tyr277Cys mutation in normal controls. M, DNA marker; P, case 10; Fa and Ma are the father and mother of case 10, respectively; N1–6 are normal controls. Case 10 and his mother carrying the p.Tyr277Cys mutation showed two products, a 251-bp fragment corresponding to the internal control and a 182-bp fragment corresponding to p.Tyr277Cys. (**C**) Alignment of the *SMN* protein. * indicates highly conserved amino acids. The colon (:) is an indicator of conserved amino acids. The period (.) indicates amino acids that are not conserved. P.Glu134 and p.Tyr277 located in the Tudor domain and Y/G box, respectively, are highly conserved.

Six subtle mutations were identified in the present study (Table 1). Five mutations (p.Ser8LysfsX23, p.Leu228X, p.Ser230Leu, p.Glu134Lys, and p.Arg288Met) were detected in more than one patient. A novel mutation (p.Tyr277Cys) located in exon 6 of *SMN1* was identified for the first time (Figure 1A). The p.Arg288Met mutation has been previously described [22]. No subtle mutations were detected in two of the patients carrying one copy of *SMN1.*

### Screening for the p.Tyr277Cys mutation

AS-PCR was performed to screen for the novel p.Tyr277Cys mutation in control individuals (Figure 1B). This mutation was not observed in the 150 control individuals. Sequence alignment of six different species showed that the Tyr277 residue was highly conserved (Figure 1C).

### *SMN* transcripts analysis of patients with subtle mutations

**Figure 2:**
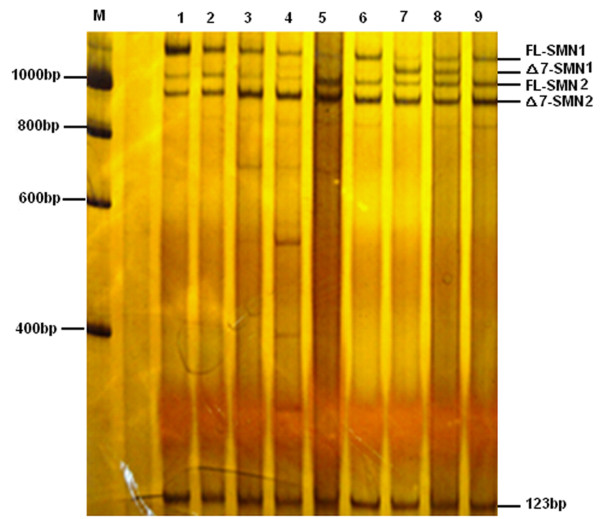
***Dde*I digestion of SMN transcripts. **M: DNA marker; lane 1: normal controls carrying two *SMN1 *and two *SMN2 *copies; 2: case 2 carrying the p.Ser8LysfsX23 mutation; 3: case 4 carrying the p.Glu134Lys mutation; 4: case 6 carrying the p.Leu228X mutation; 5: case 8 carrying the p.Ser230Leu mutation; 6: case 10 carrying the p.Tyr277Cys mutation; 7 and 8: patient 12 and her father carrying the p.Arg288Met mutation; 9: the carrier with one copy of the *SMN1 *gene. Transcripts for fl-*SMN1*, fl-*SMN2 *and Δ7-*SMN2 *were separated using 6% polyacrylamide gel electrophoresis following digestion with *Dde*I. In patient 11 carrying the p.Arg288Met mutation (line 7), the fl-*SMN1 *transcript (1259 bp) almost disappeared, but the undigested △7-*SMN1 *fragment (1205 bp) was more prominent. This was also observed in the patient’s father (line 8).

A DdeI digest of *SMN* transcripts assay was carried out to qualitative analysis the *SMN* transcript in all the patients with subtle mutations. Following *Dde*I digestion, three obvious products (fl-*SMN1,* fl-*SMN2* and Δ7-*SMN2*) were detected, without obviously truncated or prolonged transcripts in most patients (Figure 2). While in patients carrying the p.Arg288Met mutation, the fl-*SMN1* transcript (1259 bp) was rare, but the undigested Δ7-*SMN1* fragment (1205 bp) was more prominent (Figure 2). An undigested Δ7-*SMN1* fragment was also observed in their father with the p.Arg288Met mutation.

### Clone sequencing of the patients with p.Arg288Met mutation

**Figure 3:**
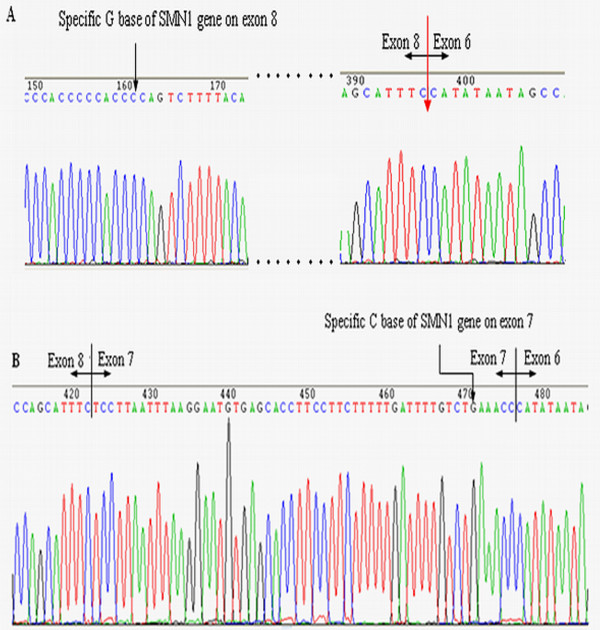
**Partial reverse-sequencing map of *SMN1 *clones for the patient with the p.Arg288Met mutation. **(**A**) Antisense sequencing map of *SMN1 *clones. The C base (G in the sense sequence) is indicated by the black arrow, which is specific for *SMN1 *on exon 8. The red arrow indicates the position of the entire exon 7 sequence that was deleted in the *SMN1 *clone. (**B**) Antisense sequencing map of a normal individual. The G base (C in the sense sequence) is indicated by the black arrow and is specific for *SMN1 *on exon 7. The intact sequence of exon 7 can be seen between exons 6 and 8.

The *SMN* cDNA (from exon 1 to exon 8) of patients with p.Arg288Met were amplified and subclone to the pGEM-T vector. After screening the *SMN1* clones and sequencing, we found that all 5 subclones of *SMN1* from these patients had lost the entire sequence of exon 7 (Figure 3), while three full-length *SMN* clones belong to the sequences of *SMN2*. This result was consistent to the result of DdeI digest of *SMN* transcripts assay. The p.Arg288Met mutation produces a transcript of Δ7-*SMN1* other than the transcript of fl-*SMN1,* it implies that it might cause the skip of exon 7 of *SMN1* gene.

### Comparison of fl-*SMN1* transcripts

**Figure 4:**
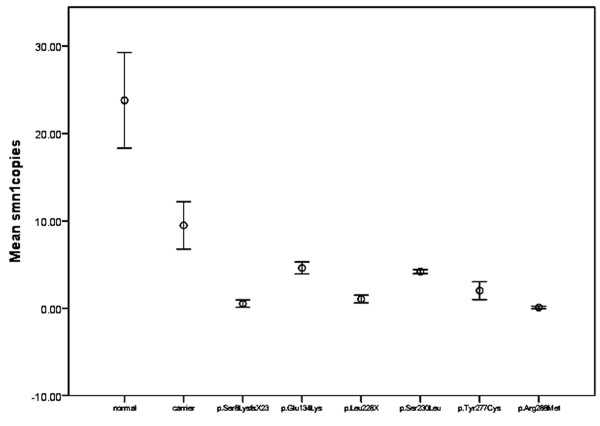
**Levels of fl-*SMN1 *transcripts in controls, carriers and patients with subtle mutations. **The dark horizontal lines indicate the 95% confidence interval. The empty circle indicate the median. Fl-*SMN1* transcript levels were significantly different between normal controls and healthy carriers. The levels of transcripts in patients were significantly reduced compared with those in controls. Fl*-SMN1 *transcript levels in patients with the p.Arg288Met mutation were almost undetectable. In patients with p.Ser8LysfsX23 or p.Leu228X mutations, transcript levels were severely reduced. In the patient carrying the novel p.Tyr277Cys mutation, transcript levels were decreased, while patients with missense mutations (p.Ser230Leu and p.Glu134Lys) showed no significantly decrease compared with carriers.

To evaluate the effect of subtle mutations on fl-*SMN1* levels, we compared fl-*SMN1* levels in patients with that of normal controls, and with healthy carriers (Table 3 and Figure 4). The fl-*SMN1* levels in normal controls and healthy carriers were 23.77 ± 7.74 and 9.47 ± 5.39, respectively. The difference between these two groups was statistically significant (t = 5.296, *P* = 0.000). The fl-*SMN1* transcript levels in all patients were significantly decreased compared with normal controls (t = 7.839, *P* = 0.000). Compared with healthy carriers carrying one copy of *SMN1*, patients with the p.Glu134Lys or p.Ser230Leu mutation showed no significant difference (t = 1.769, *P* = 0.094 and t = 1.660, *P* = 0.115, respectively), patients with the novel p.Tyr277Cys mutation presented a slight decrease in fl-*SMN1* transcript levels (t = 2.337, *P* = 0.032), while the patients with the p.Ser8LysfsX23, p.Leu228X, and p.Arg288Met mutations were significantly reduced (t test, *P* = 0.000). Especially for the patients carrying p.Arg288Met, fl-*SMN1* transcript levels were almost undetectable (0.017 ± 0.15).

**Table 3:** **Fl-*SMN1 *transcript levels of controls ,carrier , and patients with SMN1 subtle mutations**

		**Control**	**Carrier**	**p.Ser8LysfsX23**	**p.Glu134Lys**	**p.Leu228X**	**p.Ser230Leu**	**p.Tyr277Cys**	**p.Arg288Met**
n		4	8	2	2	1	2	1	2
Type				II	II	I	II,III	II	I,II
fl -*SMN1*	Mean ± SD	26.49 ± 13.88	185.71 ± 148.00	0.61 ± 0.30	6.30 ± 2.02	1.57 ± 0.38	3.54 ± 0.95	7.19 ± 2.72	0.05 ± 0.10
	Min-Max	11.16-50.8	29-470	0.22-0.95	3.58-8.26	1.24-1.98	2.94-4.64	4.90-10.20	0-0.19
*GAPDH*	Mean ± SD	2322.2 ± 1174.9	4857.3 ± 2452.5	3720 ± 1969	2742 ± 829.4	3247 ± 1079	1695 ± 456	8173 ± 2957	3002 ± 1801
	Min-Max	1232-4700	1976-8660	2000-6500	1788-3680	2040-4120	1388-2220	5340-11240	1315-5300
corrected fl -*SMN1*	Mean ± SD	23.77 ± 7.74	9.47 ± 5.39	0.52 ± 0.46	4.59 ± 0.68	1.04 ± 0.39	4.18 ± 0.20	2.01 ± 0.89	0.07 ± 0.15
	Min-Max	15.92-34.74	1.05-18.73	0.15-1.15	3.98-5.29	0.69-1.46	3.98-4.38	1.23-2.98	0-0.29
P value (t-test)		-	0.000 ^a^	0.000^b^	0.094^b^	0.000^b^	0.11^b^	0.032^b^	0.000^b^

fl -*SMN1*, *GAPDH* ,and corrected fl- *SMN1* indicate transcript levels, measured as no. of molecules per nanogram of total RNA. Superscripted a means that the t-test was carried out between controls and carriers; Superscripted b means the t-test was performed between carrier and the patients with subtle mutations.

### Transcript levels of fl-*SMN* in different clinical cases

**Figure 5:**
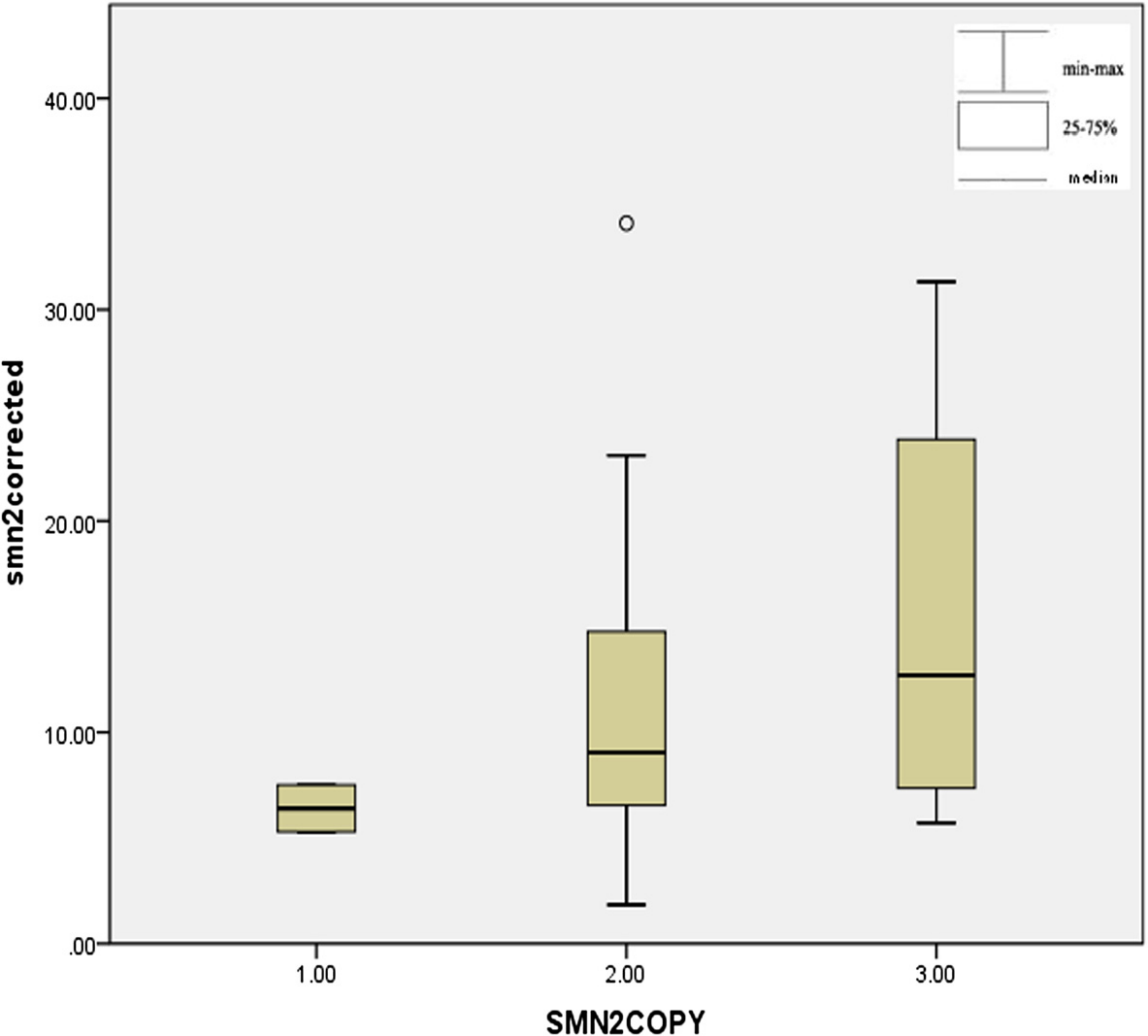
**fl-*SMN2 *transcript levels among the individuals with different *SMN2 *copy numbers. **Mean fl-*SMN2 *transcript levels in healthy individuals (normal controls and healthy carriers) with a single copy of *SMN2 *were 6.3895 ± 1.56, 11.48 ± 8.01 for those with two copies of *SMN2*, and 15.61 ± 11.39 for patients with three *SMN2 *copies. No correlation was observed between fl-*SMN2 *transcript level and *SMN2 *copy numbers (F = 0.391, *P* = 0.679).

Although there was no correlation between fl-*SMN2* transcript level and *SMN2* copy numbers (one-way ANOVA, F = 0.391, *P* = 0.679) in normal controls and healthy carriers, the fl-*SMN2* transcript level was increased along with *SMN2* copy numbers (Figure 5). The fl-*SMN2* transcript levels in patients are presented in Table 1, with no significant differences (F = 1.029, P = 0.430). Total fl-*SMN* transcript levels (fl-*SMN1* + fl-*SMN2*) were used to assess the correlation between the fl-*SMN* levels and the clinical severity. The fl-*SMN* transcript levels in normal controls and healthy carriers were 34.4 ± 7.1 and 21.7 ± 12.7, respectively, with a significant difference (t = 2.596, P = 0.017). The fl-*SMN* transcript levels in all patients were significantly decreased compared with normal controls (t = 7.060, *P* = 0.000). Mean fl-*SMN* transcript levels in type I (*n* = 2) and type II (n = 8) were 11.27 ± 6.8 and 11.67 ± 6.34, respectively. For type III (*n* = 2) patients, a slight increase in levels could be observed (20.00 ± 9.43). Because the number of types I and III patients were low, statistical analysis of fl-*SMN* transcript levels was only carried out between type II patients and healthy carriers, with a significant difference observed (t = 3.046, P = 0.009).

## Discussion

Including the results from this study, 61 *SMN1* subtle mutations have been detected among diverse populations worldwide. Nine subtle mutations have been identified in Chinese SMA patients [18-23] (Table 4). Among these, the c.1-39A > G, p.Ser8LysfsX23, p.Ser28Ser, p.Leu228X, p.Tyr277Cys, and c.835-1 G > A mutations were only found in Chinese SMA patients, p.Arg288Met mutation had been reported in another East Asian country-Korea [17], only the two mutation (p.Glu134Lys [27], and p.Ser230Leu [28]) were found in Caucasian population. In East Asian country, there was still another mutation p.Trp92Ser only reported in Japanese SMA patient [15]. These reports showed the kinds of *SMN1*subtle mutation in the Chinese even East Asian population are distinct from those in Caucasian populations. In addition, the type of common mutations differed between Chinese and Caucasian populations. In Chinese SMA patients, p.Ser8LysfsX23 and p.Leu228X seem to be the common mutations, which were detected in seven families (33.3%, 7/21) and four families (19.0%, 4/21), respectively. While in Caucasian populations, Mutations p.Arg133fsX15, p.Gly261LeufsX8, p.Tyr272Cys and p.Thr274Ile were commonly detected [4,6-8,10,11,16,25,29-31]. Our research implies that the *SMN1* subtle mutations show high heterogeneity in various populations.

**Table 4:** **Subtle mutations of *SMN1 *gene identified in Chinese SMA patients**

**Exon/ intron**	**cDNA mutation**	**Protein prediction**	**Mutation type**	**References**	**Number of families**	**Phenotype**
5′UTR	c.-39A > G	-	-	Wang CC et al. [21]	1	NA
Exon 1	c.22dupA	**p.Ser8LysfsX23**	Frameshift	Tsai et al. [18] (first report)	1	I
				Zeng J et al. [23]	1	I
				Wang CC et al. [21]	2*	NA
				This work	3	I、II
Exon 2	c.84 C > T	p.Ser28Ser	Silence	Wang CC et al. [21]	1	NA
Exon 3	c.400 G > A	p.Glu134Lys	Missense	This work	2	II
Exon 5	c.683 T > A	**p.Leu228X**	Nonsense	Tsai et al. [18] (first report)	1	I
				Zeng J et al. [23]	1	I
				This work	2	I、II
Exon 5	c.689C > T	p.Ser230Leu	Missense	Zeng J et al. [19] (first report)	1	I
				This work	1*	II、III
Exon 6	c.830A > G	p.Tyr277Cys	Missense	This work (first report)	1	II
Intron 6	c.835-1 G > A	-	Splice site	Zhu SY et al. [20]	1	I
Exon 7	c.863 G > T	p.Arg288Met	Missense	Qu YJ et al. [22]	2	I、II

*, indicate these families have two patients who were siblings. *NA*, not available; mutations in bold are common mutations in Chinese SMA patients.

Our results show that the level of fl-*SMN1* transcripts in normal controls was significantly higher than in healthy carriers, which corresponded to the different number of *SMN1* copies carried in these individuals. Although the patients with subtle mutation carried only one *SMN1* copy, the effect of these mutations on the fl-*SMN1* transcript levels were different. Based on the qPCR analysis, fl-*SMN1* transcript levels in patients with p.Ser8LysfsX23 or p.Leu228X mutations were much lower than in healthy carriers. We presumed the reason for this might be that these two premature termination mutations initiate nonsense-mediated mRNA decay (NMD). NMD procedure can result in rapid degradation of *SMN1* mRNA [32]. A lack of any significant difference between carriers and patients with missense mutations (p.Glu134Lys and p.Ser230Leu) implied that these missense mutations do not affect transcription of *SMN1*, or degradation of *SMN1* mRNA. However, all the patients with subtle mutations significantly reduced fl-*SMN* transcript levels compared with normal controls. These results were similar to those seen in patients with a homozygous deletion of *SMN1*[26]. We have attempted to assess the correlation between the fl-*SMN* levels and the clinical severity, while the difference of fl-*SMN* transcript levels in these patients were not significant. A direct relationship could not be observed between fl-*SMN* transcript levels and phenotypic severity in this study.

The p.Arg288Met mutation was firstly reported in 2009 [17], and was also found in two unrelated Chinese patients in a previous study [23]. Kang *et al.* predicted that this mutation was likely to be deleterious to protein structure and function [17]. In our study, an interesting finding was that the *SMN1* transcript carrying the p.Arg288Met mutation skipped exon 7 entirely. According to sequence analysis and restriction digestion assays, these two patients with p.Arg288Met mutation produce a transcript corresponding to Δ7-*SMN1* other than a transcript of fl-*SMN1*. Subsequent qPCR analysis verified that fl-*SMN1* transcripts were almost undetectable (0.017 ± 0.15). The effect of this mutation was similar to that seen with the C to T conversion in *SMN2* exon 7 producing Δ7-*SMN* mRNA. This transcript encodes a truncated SMN protein that fails to undergo self-oligomerization of SMN, is unstable, and degrades easily. The pathogenic effect in a patient who has only one copy of *SMN1* with the p.Arg288Met mutation is the same as for the homozygous deletion of *SMN1*. The phenotypes in patients carrying the p.Arg288Met mutation were type I SMA in the two patients and type II SMA in another patient. These results were consistent with a phenotype in SMA patients that have a homozygous deletion of *SMN1.*

Rare missense mutations were reported to affect the splicing of *SMN1*. There were two variations that occurred in exon 7 of *SMN2* that were described to affect the splicing of *SMN2*. The C→T conversion at position six of *SMN2* exon 7 was found to cause the entire exon to be skipped [33,34]. The missense mutation p.Gly287Arg (c.859 G > C) in *SMN2*, recently described as a positive disease modifier, could improve *SMN2* exon 7 inclusion [35]. Many studies have revealed that multiple cis-elements and splicing factors participate in the regulation of *SMN* exon 7 splicing. Singh *et al.*[36] showed that the Conserved tract is a positive element located in the middle of exon 7 at positions 16–44. Hofmann *et al.*[37] revealed binding of exonic splicing enhancers (ESEs) with Htra2-b1 in the middle of exon 7 at positions 19–27. In this study, our results show that the p.Arg288Met mutation may influence splicing of exon 7, causing the entire exon to be skipped. Serine/arginine-rich protein-binding ESE elements were not observed in wild-type or mutant sequences at c.863 using ESE finder 2.0 (http://rulai.cshl.edu/tools/ESE2). However, the p.Arg288Met mutation at position 29 of exon 7 was located within the Conserved tract element, and near where the ESE binds with Htra2-b1. We speculate that an unascertained ESE, regulating *SMN* exon 7 splicing, might exist in the Conserved tract element and that this variant of G > T in c.863 may influence the ESE site or produce a new exonic splicing silencer element (ESS).

The tyrosine/glycine-rich sequence (Y/G box), containing highly conserved residues Tyr268 and Gly279 (YXXGYXXGYXXG) in the C-terminus of SMN, is an essential self-oligomerization domain of SMN. It plays role in assembling of the SMN complex and participating in pre-mRNA splicing [38,39]. The aromatic amino acids Tyr268, Tyr272, and Tyr276, each at three-base intervals, seem to be more important than other amino acids in the Y/G box because two SMN monomers might form a stable dimer through aromatic stacking of these tyrosines [16]. When these three tyrosines mutate, SMN self-oligomerization could be severely disturbed. To date, several missense mutations in the Y/G box, including p.Tyr272Cys, p.His273Arg, p.Thr274Ile, p.Gly275Ser, p.Gly279Cys and p.Gly279Val, have been reported [4,7,10-14,16,40-44]. Among these, p.Tyr272Cys and p.Gly279Val were reported to be usually associated with the more severe type I form of SMA [4,11-13,44]. The p.Thr274Ile, p.Gly275Ser and p.Gly279Cys mutations were generally associated with the milder, type II and III phenotypes [7,10-13,16,41-43].

In this paper, the p.Tyr277Cys mutation within the Y/G box is reported for the first time. Although Tyr277 is an aromatic amino acid, it is near the critical Tyr276 residue. Both Tyr276 residues from two SMN monomers require adequate space to form aromatic stacking. We speculate that Tyr277 replaced by a Cys residue might disturb the space structure of aromatic stacking, thereby affecting the stability of the SMN dimer. The patient carrying the p.Tyr277Cys mutation also had two copies of *SMN2*. The mean fl-*SMN* transcript level in this patient was 6.54 ± 4.33, which was much lower than that in normal controls (34.4 ± 7.1) and healthy carriers (21.7 ± 12.7). The phenotype of this patient in our study was indicative of type II SMA. Although Tyr277 is a highly conserved residue, we predicted that the p.Tyr277Cys mutation might be a milder mutation affecting the structure of the Y/G box.

It was worth to note that two siblings (cases 8 and 9) with the same p.Ser230Leu mutation displayed different phenotypes. The elder brother was diagnosed as type II SMA, while his younger sister was type III SMA. Cases 8 and 9 carried two *SMN2* copies, with mean fl-*SMN* transcript levels of 12.22 ± 7.07 and 14.83 ± 4.45, respectively. The difference between them was not significant (t = −0.453, *P* = 0.682). We hypothesized that there might be other modifying factors that affect phenotypes, such as *Plastin 3*, which has been reported as a sex-specific protective modifier of SMA [45-47].

## Conclusion

In conclusion, six *SMN1* subtle mutations were identified in 12 patients, including the novel p.Tyr277Cys mutation. Based on our research and other studies, p.Ser8LysfsX23 and p.Leu228X mutations seem to be the common *SMN1* subtle mutations in Chinese patients. This preliminary study revealed that *SMN1* subtle mutations in Chinese SMA were different to those observed in Caucasian populations. A qRT-PCR assay implied that p.Ser8LysfsX23, p.Leu228X, and p.Arg288Met mutations affected fl-*SMN1* transcript levels. Besides, we discovered that the p.Arg288Met mutation disturbed the splicing of exon7 *SMN1* pre-mRNA, resulting in *SMN1* transcripts skipping exon 7.

## Competing interests

The authors declare no potential competing interests with respect to the authorship and/or publication of this article.

## Authors’ contributions

SF conceived of, designed and organized the study, contributed to obtaining the funding, and helped to critically revise the manuscript. QY contributed to obtaining the funding, carried out the experiments, analyzed data, and wrote the initial draft of the manuscript. DJ helped to carry out the subtle mutation analysis experiments (Cloning and sequencing); LE was responsible for diagnosis and management of patients; BJ contributed to the writing of the manuscript; JY and WH contributed to the sequencing experiments. All authors approved the final version of the manuscript submitted for publication.

## Pre-publication history

The pre-publication history for this paper can be accessed here:

http://www.biomedcentral.com/1471-2350/13/86/prepub
